# The significance and robustness of a plasma free amino acid (PFAA) profile-based multiplex function for detecting lung cancer

**DOI:** 10.1186/1471-2407-13-77

**Published:** 2013-02-15

**Authors:** Masato Shingyoji, Toshihiko Iizasa, Masahiko Higashiyama, Fumio Imamura, Nobuhiro Saruki, Akira Imaizumi, Hiroshi Yamamoto, Takashi Daimon, Osamu Tochikubo, Toru Mitsushima, Minoru Yamakado, Hideki Kimura

**Affiliations:** 1 Division of Thoracic Diseases, Chiba Cancer Center, 666-2, Nitona-cho, Chuo-ku, Chiba, 260-8717, Japan; 2 Department of Thoracic Surgery, Osaka Medical Center for Cancer and Cardiovascular Diseases, 1-3-3, Nakamichi, Higashinari-ku, Osaka, 537-8511, Japan; 3 Department of Pulmonary Oncology, Osaka Medical Center for Cancer and Cardiovascular Diseases, 1-3-3, Nakamichi, Higashinari-ku, Osaka, 537-8511, Japan; 4 Department of Anesthesia, Gunma Prefectural Cancer Center, 617-1, Takahayashi-nishicho, Ohta, 373-8550, Japan; 5 Institute for Innovation, Ajinomoto, CO., Inc, 1-1, Suzuki-cho, Kawasaki-ku, Kawasaki, 210-8681, Japan; 6 Department of Biostatistics, Hyogo College of Medicine, 1-1, Mukogawa-cho, Nishinomiya, Japan; 7 Kanagawa Health Service Association, 58, Nihon-odori, Naka-ku, Yokohama, 231-0021, Japan; 8 Department of Gastroenterology, Kameda Medical Center Makuhari, 1-3-CD2, Nakase, Mihama-ku, Chiba, 261-8501, Japan; 9 Center for Multiphasic Health Testing and Services, Mitsui Memorial Hospital, 1, Kanda-izumicho, Chiyoda-ku, Tokyo, 101-8643, Japan

**Keywords:** Plasma, Amino acid, Lung cancer, Early detection

## Abstract

**Background:**

We have recently reported on the changes in plasma free amino acid (PFAA) profiles in lung cancer patients and the efficacy of a PFAA-based, multivariate discrimination index for the early detection of lung cancer. In this study, we aimed to verify the usefulness and robustness of PFAA profiling for detecting lung cancer using new test samples.

**Methods:**

Plasma samples were collected from 171 lung cancer patients and 3849 controls without apparent cancer. PFAA levels were measured by high-performance liquid chromatography (HPLC)–electrospray ionization (ESI)–mass spectrometry (MS).

**Results:**

High reproducibility was observed for both the change in the PFAA profiles in the lung cancer patients and the discriminating performance for lung cancer patients compared to previously reported results. Furthermore, multivariate discriminating functions obtained in previous studies clearly distinguished the lung cancer patients from the controls based on the area under the receiver-operator characteristics curve (AUC of ROC = 0.731 ~ 0.806), strongly suggesting the robustness of the methodology for clinical use. Moreover, the results suggested that the combinatorial use of this classifier and tumor markers improves the clinical performance of tumor markers.

**Conclusions:**

These findings suggest that PFAA profiling, which involves a relatively simple plasma assay and imposes a low physical burden on subjects, has great potential for improving early detection of lung cancer.

## Background

Several minimally invasive, easy-to-use cancer diagnostic methods using peripheral blood samples have recently been developed to ease the physical burden on patients and to reduce cost and time [1-3]. Computer-aided systems for data mining, (e.g., using multivariate analysis) are now readily available and have shown promising results when applied to metabolic profiles for diagnostic and clinical use [4-6]. Several applications using metabolome analysis based on machine learning to diagnose human cancer using peripheral blood or urine have recently been demonstrated [7-12].

Among metabolites, amino acids are one of the most suitable candidates for focused metabolomics because they are either ingested or synthesized endogenously and play essential physiological roles both as basic metabolites and metabolic regulators. To measure amino acids, plasma free amino acids (PFAAs), which are abundant in the circulation and link all organ systems, are favorable targets because PFAA profiles are influenced by metabolic variations in specific organ systems induced by specific diseases [13-18]. Furthermore, several investigators have reported changes in PFAA profiles in cancer patients, including lung cancer patients [19-27]. However, several discrepancies exist between the results of these studies due to the limited size of the data set [22].

High-throughput techniques using high-performance liquid chromatography (HPLC)– electrospray ionization (ESI)–mass spectrometry (MS) to measure amino acids with sufficient accuracy for clinical use have also recently been developed [28-31].

By combining these technologies, we recently obtained preliminary data on the efficacy of a diagnostic index based on PFAA concentrations, known as the “AminoIndex technology”, which compresses multidimensional information from PFAA profiles into a single dimension and maximizes the differences between patients and controls. This technology was shown to be useful in the early detection of colorectal, breast, and lung cancers in approximately 150 samples from a single medical institute [32,33]. Furthermore, we also verified the efficacy and statistical robustness of this method using larger sample sizes from multiple medical institutes and developed discriminating functions to detect five types of cancer, including lung, gastric, colorectal, breast, and prostate cancer [34,35]. We also found that changes in PFAA profiles that were common to all types of cancer as well as those specific to individual cancers [34] .These functions are used in the “AminoIndex® Cancer Screening” service in Japan.

Lung cancer has been the leading cause of cancer death since 1998, and in Japan, >60,000 patients have died from lung cancer since 2005 [36]. Conventionally, chest X-rays and sputum cytology are used to screen for lung cancer in patients in Japan. However, neither chest X-rays nor sputum cytology are ideal or versatile enough to detect early lung cancer. Although chest X-rays are useful for detecting peripheral lung cancer, this method is not always suitable for early detection [37]. In addition, this technique requires highly skilled technicians to achieve sufficient accuracy. Sputum cytology has been reported to be useful only for the detection of squamous cell carcinoma and is inadequate for detecting adenocarcinoma (which is the major histological type of lung cancer in Japan) or for detecting lung cancer in asymptomatic non-smokers [37].

Compared to chest X-ray and sputum cytology, a PFAA-based diagnostic method would be easier to use because it involves a relatively simple plasma assay, imposes a lower physical burden on patients and does not require advanced technical skills. Moreover, this method can also detect lung cancer regardless of cancer stage and histological type, including small cell lung cancer [32,34,35].

In this study, we aimed to verify the usefulness of PFAA profiling for lung cancer detection using samples that had never been used as a data set to derive discriminating functions. As a result, highly reproducible results were observed in both the PFAA profiles and the discriminating performance of previously obtained PFAA-based, multiplex discriminant functions, suggesting the robustness of PFAA profiling for the early detection of lung cancer.

## Methods

### Ethics

The study was conducted in accordance with the Declaration of Helsinki, and the protocol was approved by the ethics committees of the Chiba Cancer Center, the Osaka Medical Center for Cancer and Cardiovascular Diseases, the Gunma Prefectural Cancer Center, the Kanagawa Health Service Association, the Kameda Medical Center Makuhari, and the Mitsui Memorial Hospital. All subjects gave their written informed consent for inclusion before participating in the study. All data were analyzed anonymously throughout the study.

### Subjects

The participants in this study consisted of Japanese patients who had previously been histologically diagnosed with lung cancer at the Chiba Cancer Center (n=171) between 2007 and 2009. Control subjects (n=3849) without apparent cancers who were undergoing comprehensive medical examinations at the Kanagawa Health Service Association, the Kameda Medical Center Makuhari, or the Mitsui Memorial Hospital, Japan between 2008 and 2010 were recruited to participate in the study. Among the participants, 85 cancer patients (P1) and 421 gender- and age-matched controls (C1) were used as the study dataset for two preliminary studies (Table 1) [32,34]. The remaining 86 cancer patients (P2) and 323 gender- and age-matched controls (C2) were used as a test dataset and were not used to derive the discriminating functions in previous studies (Table 1) [32,34]. The remaining 3427 unmatched controls (C3) were also included and were not used to derive the discriminating functions in previous studies (Table 1) [32,34].

**Table 1:** Demographic and clinical characteristics of the subjects

**Subjects**		**Patients**		**Controls**		
**Subgroup**		**P1**	**P2**	**C1**	**C2**	**C3**
Dataset	1	Used		Used		
	2		Used		Used	
	3	Used	Used	Used	Used	Used
	4	Used	Used			
Number	Total	85	86	421	323	3104
	(Male, Female)	(49,36)	(68,18)	(245,176)	(263,60)	(1898,1206)
Age, y	Mean (SD)	65.1 (9.7)	67.8 (8.2)	63.1 (8.7)	61.9 (6.0)	49.4 (8.0)
	Range	30-90	41-83	28-86	37-88	23-67
BMI	Mean (SD)	22.1 (3.7)	22.4 (3.2)	22.8 (3.0)	23.4 (2.9)	23.2 (3.3)
	Range	14.6~31.2	15.7-34.6	14.2-37.1	16.9-35.4	14.8-41.2
Smoking status	Never	26	18	222	139	1865
	Ex	29	36	106	107	434
	Current	29	29	57	62	695
	Unknown	1	3	36	15	110
pStage^a^	I	33	33			
	II	5	5			
	III	27	22			
	IV	20	23			
	Unknown	0	3			
Histology	Adenocarcinoma	59	55			
	Squamous cell carcinoma	13	12			
	Other NSCLC	5	8			
	SCLC	8	11			

a: Cancer stages were determined according to the International Union Against Cancer TNM Classification of Malignant Tumors, 6th Edition [38].

Using these subjects, four data sets were evaluated in this study. Dataset 1 includes P1 and C1, Dataset 2 includes P2 and C2, Dataset 3 includes all of the subjects involved in this study (P1, P2, C1, C2, and C3), and Dataset 4 includes all of the patients involved in this study (P1 and P2) (Table 1).

### Measurement of plasma amino acid concentration

Blood samples (5 ml) were collected from forearm veins, after overnight fasting, in tubes containing ethylenediaminetetraacetic acid (EDTA; Termo, Tokyo, Japan) and were immediately placed on ice. Plasma was prepared by centrifugation at 3,000 rpm and 4°C for 15 min and stored at −80°C until analysis. The plasma samples were deproteinized using acetonitrile at a final concentration of 80% before measurement. The amino acid concentrations in the plasma were measured by HPLC–ESI–MS followed by precolumn derivatization. The analytical methods used have previously been described [29-31]. Among the 20 genetically encoded amino acids, glutamate (Glu), aspartate (Asp), and cysteine (Cys) were excluded from the analysis because they are unstable in blood. Citrulline (Cit) and ornithine (Orn) were measured instead because they are relatively abundant in blood and are known to play important roles in metabolism. The following 19 amino acids were measured and analyzed: alanine (Ala), arginine (Arg), asparagine (Asn), Cit, glutamine (Gln), glycine (Gly), histidine (His), isoleucine (Ile), leucine (Leu), lysine (Lys), methionine (Met), Orn, phenylalanine (Phe), proline (Pro), serine (Ser), threonine (Thr), tryptophan (Trp), tyrosine (Tyr), and valine (Val). The concentrations of amino acids in the plasma were expressed as μM values. For analysis of the PFAA profile, two measurements were conducted for each of the 19 amino acids. The absolute concentration of each amino acid and the ratios of the amino acid concentrations expressed by the follow equation as previously described were used [32,34]. The concentrations of the amino acids in the plasma were expressed in μM, and the ratios of the amino acid concentrations were expressed by the follow equation:

(1) $$X2_{i,j}=\frac{X_{i,j}}{\sum_{k}\;X_{i,k}}$$

where *X2*_*i,j*_ is the ratio of the amino acid concentration of the j-th amino acid of the i-th subject, and *X*_*i,j*_ is the plasma concentration (μM) of the j-th amino acid of the i-th subject.

### Measurement of tumor markers

Using serum samples from lung cancer patients, the levels of the following five tumor markers were measured: CEA (chemiluminescence immunoassay, normal range ≦ 5.0 ng/ml), CYFRA (electrochemiluminescence immunoassay, normal range ≦ 3.5 ng/ml), ProGRP (enzyme-linked immunoadsorbent assay, normal range ≦ 46 pg/ml), SCC (enzyme immunoassay, normal range ≦ 1.5 ng/ml), and NSE (radioimmunoassay, normal range ≦ 10 ng/ml) [39].

### Calculation of discriminant scores

The PFAA profiles of subjects were substituted into the discriminating functions obtained from the results of three independent preliminary studies [32,34,35]. Both Discriminant- 1 and Discriminant- 3 were logistic regression functions, whereas Discriminant- 2 was a linear discriminating function using plasma concentrations (expressed in μM) as explanatory variables.

### Statistical analysis

#### Mean and SD

The mean amino acid concentrations ± standard deviations (SD) were calculated to determine the overall PFAA profiles for both patients and controls.

#### Mann–Whitney U-test

The Mann–Whitney *U*-test was used to evaluate differences in the PFAA profiles between the patient and control samples.

#### ROC curve analysis

Receiver-operator characteristic (ROC) curve analyses were performed to determine the abilities of both the PFAA concentrations and discriminating scores to discriminate between patients and controls. The patient labels were fixed as positive class labels. The 95% confidence interval (95% CI) for the AUC of ROC for the discrimination of patients based on amino acid concentrations and ratios was also estimated as described by Hanley and McNeil [40].

#### Pearson’s correlation coefficients

Pearson’s correlation coefficients were calculated among three kinds of discriminant scores (obtained from Discriminant- 1, Discriminant- 2, and Discriminant- 3) using Dataset 3. In addition, coefficients were also calculated using stratified data (patients and controls).

#### Determination of sensitivity

The cutoff value for Discriminant- 3 was previously determined so that 95% specificity would be obtained [35]. The sensitivity of Discriminant- 3 was also calculated as the ratio of true positives to the summation of the true positives and false negatives. For tumor markers, sensitivities were also determined as the ratio of the number of subjects in which the marker levels were higher than the previously determined normal range to the number of measured subjects.

#### McNemar test

The McNemar test was performed to evaluate the improvement in sensitivities through combinatorial use of both Discriminant- 3 and the tumor markers.

#### Software

All of the analyses were performed using MATLAB (The Mathworks, Natick, MA) and GraphPad Prism (GraphPad Software, La Jolla, CA).

## Results

### Characteristics of the patients and control subjects

Table 1 summarizes the characteristics of the subjects in this study. No significant differences in body mass index (BMI) were observed between patients and matched controls (Table 1). Weight loss due to malnutrition was therefore not expected to influence the results. Although significant differences in average age were observed between the data sets, the effects appeared to be relatively minor because the absolute values of these differences were small (Table 1). Disease stages were determined according to the Sixth Edition of the International Union Against Cancer (UICC) Tumor–Node–Metastasis (TNM) Classification of Malignant Tumors [38]. The fractions of patients at each stage according to the type of cancer were as follows: ~40% stage I, ~5% stage II, ~30% stage III, and ~25% stage IV (Table 1).

The cancer patients were also further subdivided based on histological tumor type; approximately 65% of the patients were classified as having adenocarcinoma, 15% as having squamous cell carcinoma, and 10% as having small cell lung cancer (SCLC) (Table 1).

### PFAA profiles of lung cancer patients

**Figure 1:**
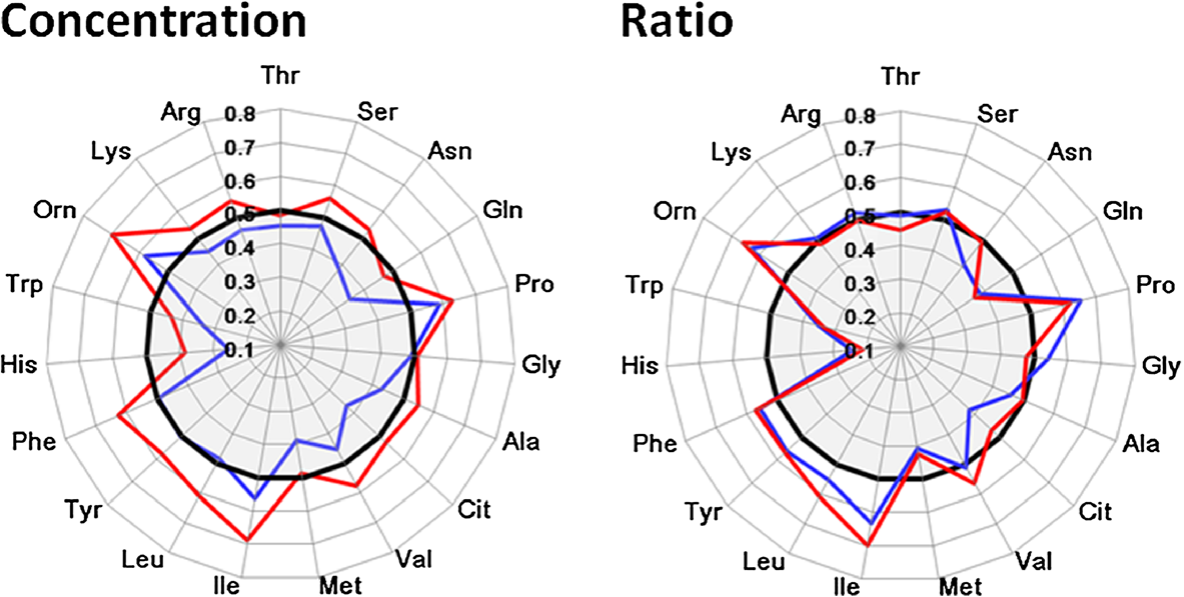
**PFAA profiles of lung cancer patients.** Axes show the AUC of the ROC for each amino acid to discriminate lung cancer patients from controls. Black bold lines indicate the point at which the AUC of the ROC = 0.5.

First, the PFAA profiles of the study data set in previous studies and of the test data set, which was never used for analysis, were used to verify the changes in PFAA profiles observed in cancer patients. Interestingly, the PFAA profiles of the test data set were quite similar to those of the study data set, especially for the ratios of the amino acid concentrations (Figure 1 and Table 2), indicating that the alteration in PFAA profiles observed in cancer patients is robust. Significant increases in both the concentration and ratio of Pro and Orn and significant decreases in His were observed in both the study and test data sets compared to controls (Figure 1 and Table 2). The ratios more clearly reflected the alterations in the PFAA profile than the concentrations; the profiles of five additional amino acids were altered in the ratio data (Gln, Met, and His were decreased in patients, and Ile was increased in patients), while significant changes in concentration were detected in only one direction (Figure 1 and Table 2).

**Table 2:** PFAA profiles of controls and lung cancer patients

**A. Concentration**												
**Amino acids**	**P1**		**C1**				**P2**		**C2**			
	**Mean**	**SD**	**Mean**	**SD**	**AUC**^**a**^		**Mean**	**SD**	**Mean**	**SD**	**AUC**^**a**^	**p**^**b**^
Thr	115.8	30.8	118.5	23.6	0.453		122.6	32.0	121.8	25.8	0.483	
Ser	107.8	20.8	108.8	18.1	0.472		110.7	20.1	106.7	17.4	0.560	
Asn	42.6	7.3	45.2	6.5	0.383	p<0.001	47.9	11.2	46.1	6.5	0.533	
Gln	547.4	71.4	586.9	64.2	0.347	p<0.001	577.5	84.1	587.1	61.5	0.470	
Pro	141.6	37.1	132.3	38.4	0.588	p<0.05	157.3	48.5	138.6	38.5	0.630	p<0.001
Gly	214.3	66.2	209.7	52.2	0.490		208.0	63.5	203.5	47.9	0.511	
Ala	324.2	84.5	343.1	74.8	0.428	<p0.05	366.5	99.8	353.5	69.7	0.550	
Cit	29.0	8.8	33.0	7.4	0.367	p<0.001	33.3	10.3	32.4	7.1	0.530	
Val	215.1	43.1	220.5	39.7	0.453		242.1	43.2	230.0	37.2	0.578	p<0.05
Met	24.2	5.4	25.8	4.6	0.388	p<0.01	26.9	7.2	26.8	4.1	0.489	
Ile	64.7	16.2	60.8	14.4	0.563		73.9	14.0	64.4	13.1	0.689	p<0.001
Leu	117.6	25.3	118.8	23.8	0.484		135.2	26.3	125.2	21.3	0.610	p<0.01
Tyr	65.9	15.0	65.2	12.5	0.503		71.9	15.9	67.3	10.9	0.579	p<0.05
Phe	59.5	9.9	59.6	9.4	0.496		66.5	12.8	61.3	7.9	0.625	p<0.001
His	69.7	12.5	80.2	9.5	0.255	p<0.001	74.1	15.8	81.2	9.4	0.383	p<0.001
Trp	51.3	11.0	57.0	8.8	0.338	p<0.004	56.4	13.4	59.6	8.9	0.432	
Orn	55.2	13.2	51.7	12.6	0.581	p<0.05	61.7	16.4	51.7	10.4	0.696	p<0.001
Lys	183.9	32.7	189.1	30.4	0.450		195.7	37.7	191.6	27.7	0.535	
Arg	93.1	20.7	95.1	16.8	0.460		100.4	24.7	96.4	15.1	0.551	
**B. Ratio**												
Thr	4.556	0.887	4.544	0.706	0.488		4.460	0.749	4.590	0.781	0.445	
Ser	4.301	0.765	4.200	0.669	0.528		4.080	0.601	4.044	0.617	0.521	
Asn	1.692	0.218	1.740	0.197	0.408	p<0.01	1.749	0.235	1.742	0.194	0.494	
Gln	21.800	2.097	22.661	2.233	0.383	p<0.001	21.276	2.079	22.253	2.053	0.364	p<0.001
Pro	5.599	1.246	5.049	1.225	0.654	p<0.001	5.756	1.504	5.213	1.211	0.622	p<0.001
Gly	8.543	2.566	8.102	1.994	0.542		7.608	1.872	7.703	1.734	0.477	
Ala	12.715	2.141	13.123	2.156	0.459		13.253	2.380	13.321	2.092	0.499	
Cit	1.150	0.328	1.278	0.288	0.378	p<0.001	1.211	0.311	1.229	0.263	0.469	
Val	8.530	1.294	8.454	1.109	0.510		8.925	1.267	8.696	1.198	0.563	
Met	0.955	0.128	0.990	0.115	0.407	p<0.01	0.979	0.152	1.011	0.108	0.424	p<0.05
Ile	2.558	0.519	2.321	0.405	0.631	p<0.001	2.727	0.441	2.427	0.415	0.698	p<0.001
Leu	4.657	0.760	4.547	0.643	0.551		4.986	0.795	4.731	0.671	0.607	p<0.01
Tyr	2.616	0.495	2.503	0.368	0.560		2.644	0.455	2.543	0.335	0.567	
Phe	2.374	0.370	2.295	0.291	0.557		2.470	0.492	2.322	0.272	0.572	p<0.05
His	2.769	0.395	3.091	0.291	0.245	p<0.001	2.702	0.355	3.074	0.295	0.213	p<0.001
Trp	2.034	0.348	2.197	0.301	0.351	p<0.001	2.057	0.373	2.260	0.313	0.338	p<0.001
Orn	2.195	0.482	1.984	0.413	0.631	p<0.001	2.298	0.674	1.955	0.353	0.662	p<0.001
Lys	7.278	0.809	7.265	0.834	0.507		7.169	0.760	7.241	0.824	0.485	
Arg	3.680	0.568	3.657	0.525	0.519		3.650	0.550	3.645	0.473	0.494	

a: AUC indicates AUC of the ROC curve.

b: *p* value derived from the Mann–Whitney *U*-test.

### Verification of multivariate discriminating functions

We used three different discriminating functions to distinguish lung cancer patients from controls (Table 3). Discriminant 1 was derived from the PFAA profiles of cancer patients recruited from the Osaka Medical Center for Cancer and Cardiovascular Diseases and controls recruited from the Center for Multiphasic Health Testing and Services of the Mitsui Memorial Hospital [32]. Discriminant 2 and Discriminant 3 were derived from patients from the Osaka Medical Center for Cancer and Cardiovascular Diseases, the Chiba Cancer Center, the Kanagawa Cancer Center, and the Gunma Prefectural Cancer Center and controls recruited from the Center for Multiphasic Health Testing and Services of the Mitsui Memorial Hospital, the Kameda Medical Center Makuhari, and the Kanagawa Health Service Association [34,35]. Discriminant 3 is commercially used in the “AminoIndex® Cancer Screening” service in Japan (Ajinomoto, CO., Inc.) [35]. Both Discriminant 1 and Discriminant 3 were logistic regression models, whereas Discriminant 2 was a linear discriminating function. Explanatory variables used in these functions are listed in Table 3.

**Table 3:** Three discriminating functions and amino acids used in each function

**Discriminant**	**Amino acids incorporated into the model**	**Reference**
1	Ala, Val, Ile, His, Trp, Orn	[32]
2	Ser, Gln Pro, Cit, Val, Ile, Phe, His, Trp, Orn	[34]
3	Ser, Gln, Ala, His, Orn, Lys	[35]

**Figure 2:**
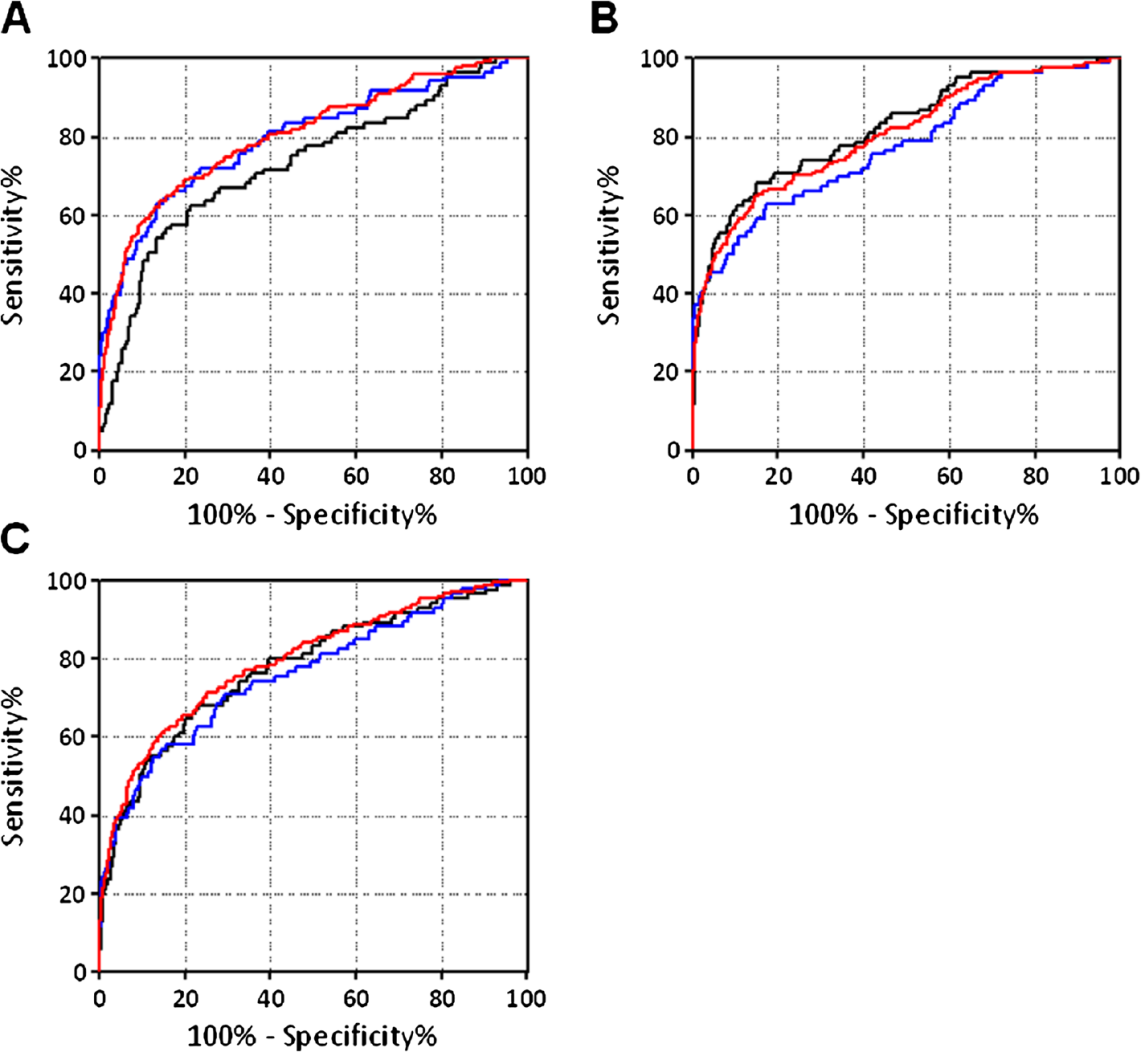
**ROC curves of discriminating scores for each discriminating function.** Black lines indicate the ROC curves of Dataset 1, blue lines indicate those of Dataset 2, and red lines indicate those of Dataset 3.

Three different data sets (Dataset 1, Dataset 2, and Dataset 3) were used to verify the performance of the discriminating functions (Table 4 and Figure 2). Notably, the discrimination abilities of each data set were evaluated using the AUC of the ROC of the discriminate score and were found to be > 0.7 in all cases, indicating that the discrimination functions were both reproducible and robust using independent data sets (Figure 2, Table 4). Specifically, AUCs for the discrimination of lung cancer patients were estimated as follows: 0.731 (95% CI: 0.668 - 0.794) for Dataset 1, 0.822 (95% CI: 0.768 - 0.875) for Dataset 2, and 0.777 (95% CI: 0.718 - 0.836) for Dataset 3 for Discriminant- 1; 0.797 (95% CI: 0.738 - 0.856) for Dataset 1, 0.775 (95% CI: 0.714 - 0.836) for Dataset 2, and 0.761 (95% CI: 0.700 - 0.823) for Dataset 3 for Discriminant 2; and 0.805 (95% CI: 0.767 - 0.846) for Dataset 1, 0.806 (95% CI: 0.767 - 0.843) for Dataset 2, and 0.795 (95% CI: 0.755 - 0.831) for Dataset 3 for Discriminant 3 (Figure 2, Table 4).

**Table 4:** AUCs of the ROC and the 95% confidential intervals (95% CIs) for each model

	**Discriminant-1**		**Dinscriminant-2**		**Discriminant-3**	
	**AUC**	**95% CI**	**AUC**	**95% CI**	**AUC**	**95% CI**
Dataset 1	0.731	0.668-0.794	0.822	0.768-0.875	0.777	0.718-0.836
Dataset 2	0.797	0.738-0.856	0.775	0.714-0.836	0.761	0.700-0.823
Dataset 3	0.805	0.767-0.846	0.806	0.767-0.843	0.795	0.755-0.831

Selected explanatory variables partially overlapped for the discriminating functions (Table 3); therefore, the discriminant scores were highly mutually correlated as presented in Table 5. The correlation coefficients were as follows: 0.609 (Discriminant- 1 and Discriminant- 2), 0.552 (Discriminant- 1 and Discriminant- 3), and 0.719 (Discriminant- 2 and Discriminant- 3) for all of the data in Dataset 3. For the patients in Dataset 3 (i.e., P1 and P2), the correlation coefficients were 0.559 (Discriminant- 1 and Discriminant- 2), 0.506 (Discriminant- 1 and Discriminant- 3), and 0.686 (Discriminant- 2 and Discriminant- 3), and the correlation coefficients for the controls in Dataset 3 (i.e., C1, C2, and C3) were 0.674 (Discriminant- 1 and Discriminant- 2), 0.645 (Discriminant- 1 and Discriminant- 3), and 0.810 (Discriminant- 2 and Discriminant- 3) (Table 5).

**Table 5:** Correlation coefficients among the discriminant scores of Dataset 3 and its subgroups obtained from three discriminating functions

**Compared with;**	**All data**	**Patients (P1, P2)**	**Controls (C1, C2, C3)**
Discriminant- 1 and Discriminant- 2	0.609	0.559	0.674
Discriminant- 1 and Discriminant- 3	0.552	0.506	0.645
Discriminant- 2 and Discriminant- 3	0.719	0.685	0.810

### Combinatorial use of discriminating functions and tumor markers

**Figure 3:**
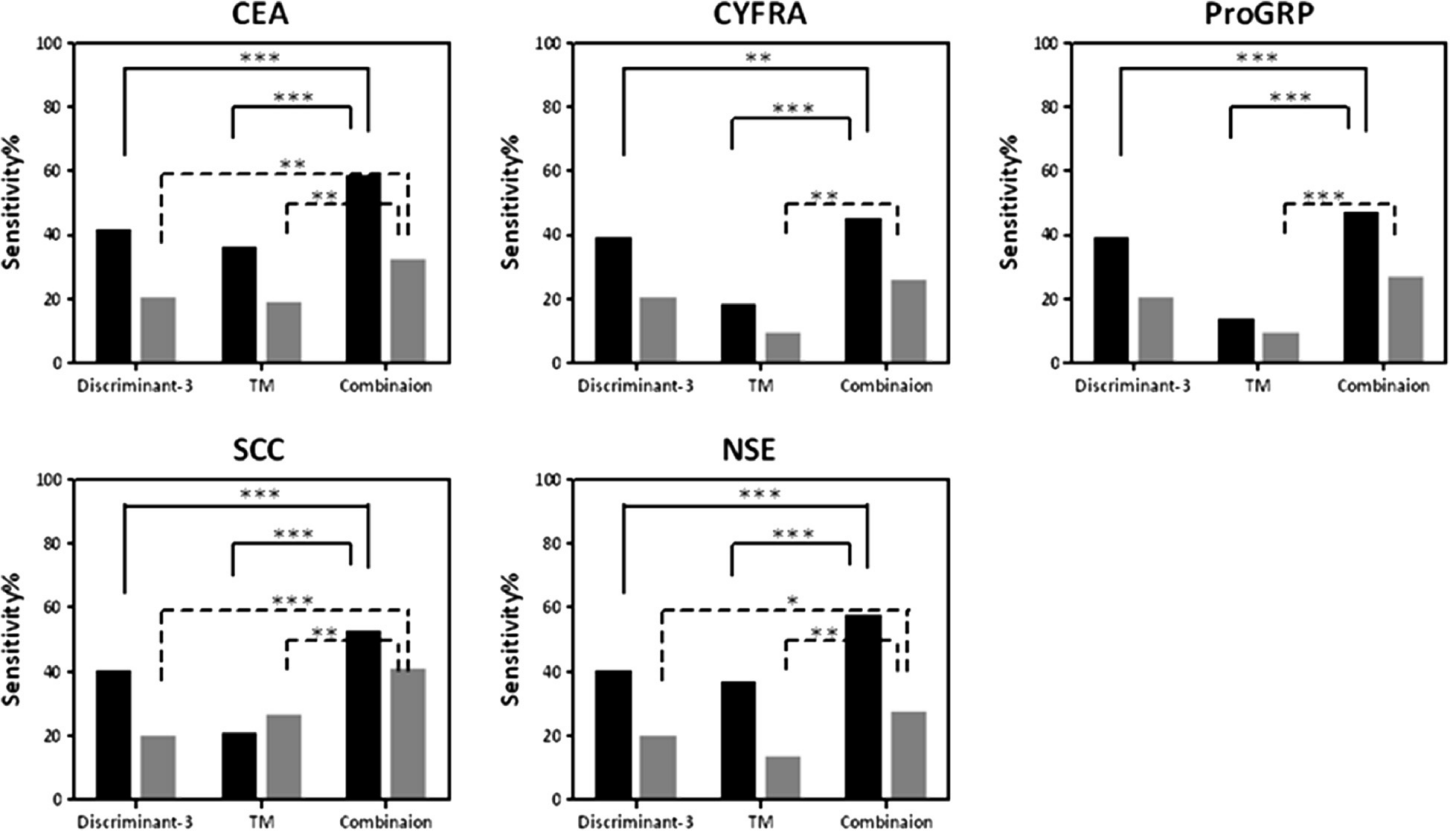
**Sensitivities of discriminating scores for Discriminant- 3, levels of tumor markers, and combinatorial use of both markers.** Black bars indicate the sensitivities of all of the data from Dataset 4, while gray bars indicate those of patients with stage I and II disease. * : p<0.05, **: p<0.01, ***: p<0.001 significant at McNemar test.

For further investigation of the clinical applicability of PFAA profiles, the combinatorial use of both the discriminating function from PFAA profiles as explanatory variables and existing tumor markers generally used for lung cancer detection and monitoring (CEA, CYFRA, ProGRP, SCC, and NSE) was assessed [39,41]. In this analysis, Dataset 4 (P1 and P2) was analyzed using discriminant scores obtained from Discriminant- 3. Subgroup analysis was also performed using patient data stratified into cancer stages (stages I and II). For all patients, significantly higher sensitivities were observed upon combinatorial use of Discriminant- 3 and the tumor markers than upon single use of either Discriminant- 3 or the tumor markers (Figure 3). Similar results were observed among stage I and II patients using the combination of Discriminant- 3 and three tumor markers (CEA, SCC, and NSE), while no significant improvement of sensitivity was observed using Discriminant- 3 and CYFRA or ProGRP (Figure 3). These results suggest that the combinatorial use of Discriminant 3 and other tumor markers is effective for lung cancer detection and monitoring, and an increase in sensitivity was indeed confirmed (Figure 3).

Among the tumor markers, CYFRA and SCC are specific to squamous cell carcinoma (SqCC), ProGRP and NSE are specific to small cell lung cancer (SCLC), and CEA is not specific to any particular histological type of lung cancer [39]. Clinically, the combinatorial use of multiple independent tumor markers is effective for detecting lung cancer. Notably, a low correlation was observed between Discriminant 3 and the tumor markers; the correlation coefficients were 0.304 for CEA, 0.481 for CYFRA, -0.228 for ProGRP, 0.346 for SCC, and 0.102 for NSE (data not shown).

## Discussion

In the present study, we verified the usefulness of PFAA profiling for lung cancer detection using new independent samples that had never been used for previous analysis and a derivation of multivariate discriminating function(s) that could distinguish lung cancer patients from control subjects. The results were highly reproducible for the change in PFAA profiles in lung cancer patients and highly discriminatory for lung cancer patients, including those with early stage cancer. Therefore, the results strongly suggest that our method is robust enough for clinical use. Moreover, because our method is a relatively simple plasma assay and imposes minimal physical burden on subjects, our findings suggest that PFAA profiling has great potential for improving the early detection of lung cancer.

Among the three discriminating functions, several amino acids were used in more than one function. His and Orn were incorporated into all of the functions, and Ser, Gln, Ala, Val, Ile, and Trp were incorporated into two of the three functions (Table 3) [32,34,35]. According to a comparison between the study and test data sets, plasma concentrations of Pro, Ile and Orn were higher in each data set, while the concentrations of Gln, His and Trp were lower (Figure 1 and Table 2). Among these amino acids, changes in the plasma concentrations of four amino acids (Pro, Ile, His, and Orn) were identical to the changes in amino acids in lung cancer patients in previous studies. Maeda et al. have reported that plasma concentrations of Ser, Pro, Gly, Ala, Met, Ile, Leu, Tyr, Phe, Orn, and Lys are increased and His is decreased in lung cancer patients [32]. Miyagi et al. have also reported that the plasma concentrations of Ser, Pro, Gly, Ile, and Orn are increased, whereas Gln, Cit, His, and Trp are decreased in lung cancer patients [34]. Therefore, the results strongly suggest the robustness of these three discriminating functions for the detection of lung cancer.

Moreover, Miyagi et al. have also reported that plasma levels of Gln, Trp, His, Pro, and Orn are commonly altered in cancer patients with five types of cancer (lung cancer, gastric cancer, colorectal cancer, breast cancer, and prostate cancer) [34]. Therefore, the data also strongly suggest that the changes in plasma concentrations of Pro, His, and Orn are essentially associated with carcinogenesis and cancer progression regardless of the location of the tumor.

Although tumor markers have been used extensively to detect lung cancer and estimate clinical condition, the markers are not always useful due to low specificity and insufficient sensitivity. Therefore, combinatorial use of two or more independent tumor markers is necessary for clinical utility [39]. Our results suggest that a PFAA-based diagnostic method would be a novel index to improve the insufficient clinical performance of the tumor markers. Combinatorial use of the tumor markers with Discriminant- 3 showed higher sensitivities than any of the tumor markers generally used for lung cancer patients. Additionally, only a low correlation was observed between the discriminating function scores and the tumor marker levels, suggesting the independence of the PFAA profiles from the existing tumor markers. Miyagi et al. have suggested that the change in the PFAA profile in cancer patients reflects two aspects: metabolic changes common to many cancers and metabolic characteristics specific to each cancer [34]. Indeed, although the results were preliminary, the same study demonstrated the possibility of discriminating the cancer type. To clarify this hypothesis, testing the behavior of the discriminating function scores in lung cancer patients after surgery and chemotherapy and in those with recurrence would be necessary.

Because this study was designed as a case–control study, the results cannot be directly applied to further observation or prediction. Therefore, additional validation using a larger sample size is necessary to establish the clinical utility of our approach. Nonetheless, we believe that our results strongly suggest the clinical usefulness of the PFAA-based diagnostic method for the detection of lung cancer.

## Competing interest

Takashi Daimon, Osamu Tochikubo, and Minoru Yamakado have been consultants for Ajinomoto, Co., Inc. and receive consultancy fees from Ajinomoto, CO., Inc. Akira Imaizumi and Hiroshi Yamamoto are employees of Ajinomoto, CO., Inc. Masato Shingyoji, Toshihiko Iizasa, Masahiko Higashiyama, Fumio Imamura, Nobuhiro Saruki, Osamu Tochikubo, Toru Mitsushima, Minoru Yamakado, and Hideki Kimura have received research grants from Ajinomoto, CO., Inc. Masahiko Higashiyama, Fumio Imamura, Akira Imaizumi, and Hiroshi Yamamoto have applied for patents for plasma amino acid profiling using multivariate analysis as a diagnostic tool for lung cancer (WO2008/016111).

## Authors’ contributions

AI, HY and HK designed this study. MS, TI, MH, FI, NS, and HK coordinated the study and collected the background data on the patients. HY, OT, TM, and MY also coordinated the study and supervised the collection of control subjects. MS and AI provided data analysis and wrote the manuscript. AI and TD performed statistical analyses. All authors read and approved the final paper.

## Pre-publication history

The pre-publication history for this paper can be accessed here:

http://www.biomedcentral.com/1471-2407/13/77/prepub
